# Combination of four gene markers to detect circulating tumor cells in the peripheral blood of patients with advanced lung adenocarcinoma using real-time PCR

**DOI:** 10.3892/ol.2013.1180

**Published:** 2013-02-05

**Authors:** YAN YU, GANG XU, JINGYAN CAO, SHI JIN, YINGCHUN MAN, LIHUA SHANG

**Affiliations:** 1 Department of Medical Oncology, The Third Affiliated Hospital of Harbin Medical University, Harbin 150080, P.R. China; 2 Department of Medical Oncology, General Hospital of Heilongjiang, Land Reclamation Bureau, Harbin 150080, P.R. China

**Keywords:** circulating tumor cells, quantitative real-time PCR, survivin, hTERT, CK-7, TTF-1

## Abstract

The aim of this study was to establish a robust and reliable assay for the detection of circulating tumor cells (CTCs) in the peripheral blood (PB) of patients with advanced lung adenocarcinoma. We used real-time reverse transcription PCR (RT-PCR) to detect survivin, human telomerase reverse transcriptase (hTERT), cytokeratin-7 (CK-7) and thyroid transcription factor 1 (TTF-1) mRNA expression levels in 68 advanced lung adenocarcinoma patients and 30 healthy patients. Statistical analyses were additionally performed to examine the correlation between the mRNA expression levels of these markers with the clinicopathological features of advanced lung adenocarcinoma patients. The sensitivity of these four mRNA markers in the PB of advanced lung adenocarcinoma patients was 41.18, 61.76, 41.18 and 35.29%, respectively. The sensitivity of these four markers combined was 82.35%, which was significantly higher compared with single marker detection. Statistical analysis demonstrated that high expression levels of survivin, hTERT and TTF-1 mRNA are positively correlated with lymph node classification, and high expression levels of survivin, hTERT, CK7 and TTF-1 mRNA are positively correlated with distant metastasis (P<0.05). In addition, overexpression of these four mRNA markers is positively correlated with disease progression (P<0.05). Our data suggest that the combination of survivin, hTERT, CK-7 and TTF-1 mRNA markers may provide a valuable tool for CTC detection and is associated with disease progression in advanced lung adenocarcinoma patients.

## Introduction

Lung cancer remains the leading cause of cancer mortality in a number of countries worldwide and non-small cell lung cancer (NSCLC) accounts for 80% of all lung malignancies (1). Lung adenocarcimoma is one of the most common subtypes of NSCLC, with high mortality rates and poor prognosis (2). The majority of lung adenocarcimoma cases present with a small primary tumor with an increased tendency to metastasize to regional lymph nodes and distant organs. Although significant advances in the diagnosis and therapy of lung cancer have been made in recent decades, the survival rate of this malignant disease has only minimally improved. Lung cancer is a multi-step process with morphological progression involving multiple molecular events (2). Thus, the identification of molecular and biological alterations that occur during carcinogenesis and progression may facilitate the investigation of the pathology of the disease and generate new markers to more accurately predict and monitor patients’ clinical outcome and therapy, subsequently aiding to individualize the treatment of cancer in patients.

Circulating tumor cells (CTCs), which are derived from primary sites or metastases, are capable of circulating freely in the peripheral blood (PB) of cancer patients (3). CTCs have been isolated and characterized in a variety of human solid tumors, including breast cancer (4), melanoma (5), gastrointestinal cancer (6), lung cancer (7) and prostate cancer (8). Evidence from previous studies highlighted that the presence of CTCs may reflect the tumor burden and is associated with tumor relapse and progression (5,9–11). Improvements in molecular technology have enabled the detection of rare CTCs in PB samples using quantification methods, including real-time PCR techniques. The main advantage of this approach lies in its sensitivity, which is considered to be higher than the reported sensitivity of immune-mediated detection, including immunocytochemistry (12). Given the heterogeneity of metastatic tumor cells, it is unlikely that one marker suitable for the detection of CTCs in all cancer patients exists. Therefore, we suggest that a multimarker assay is likely to provide an improved strategy compared with single marker assays and real-time reverse transcription PCR (RT-PCR) is easily adaptable to multimarker assays (13).

Survivin is a member of the family of inhibitors of the apoptotic proteins and has been implicated in the regulation of cell survival and mitosis in cancer (14–16). High levels of survivin expression have been detected in cancer cells, with low levels of expression detected in the majority of normal differentiated adult tissues (14). Previous studies suggest that the overexpression of survivin is correlated with advanced disease, accelerated time to recurrence, reduced survival and resistance to therapy (17). Numerous studies have demonstrated that the detection of survivin expression in CTCs is significantly correlated with disease diagnosis and clinical prognosis (18–20). The expression of the human telomerase reverse transcriptase (hTERT) gene functions as the major limiting factor for telomerase activity, which is important in cellular immortalization, tumorigenesis and the progression of cancer (21). The telomerase enzyme complex consists of two major subunits and its expression is mainly regulated by the catalytic subunit, hTERT (22). The cancer-related gene, hTERT, has been used for the detection of CTCs in the PB of breast and gastric cancer patients (20,23). Thyroid transcription factor 1 (TTF-1), which is a 38-kDa nuclear protein member of the NKx2 family of homodomain transcription factors, is selectively expressed in the lung, thyroid and diencephalons (24). It has been used to identify primary lung adenocarcinoma or thyroid tumors (25,26). Epithelial tumors usually retain the keratin expression profile of their normal epithelial origin. Cytokeratin-7 (CK-7) is a type II cytoskeletal keratin that is frequently expressed in the epithelial tissue lining the cavities (27). It has been demonstrated that CK-7 is frequently expressed in a variety of human tumors, including lung (27), gallbladder (28), breast (29), gastrointestinal (30) and urinary tract carcinoma (31). A previous study demonstrated that CK-7 may serve as a potential marker for the detection of CTCs in lung cancer (32).

In this study, we aimed to develop a real-time RT-PCR assay to investigate the expression of four epithelial or cancer-related mRNA markers, survivin, hTERT, CK-7 and TTF-1, in order to detect levels of CTCs in PB samples of advanced lung adenocarcinoma patients as well as from healthy controls. We additionally evaluated the efficacy of each individual circulating mRNA marker and all four mRNA markers in combination. Furthermore, we investigated the correlation between the expression levels of these four mRNA markers with the clinicopathological features of advanced lung adenocarcinoma patients. Lastly, we analyzed the correlation between CTC status with patients’ disease progression.

## Materials and methods

### Patient and control sample selection

Sixty-eight lung adenocarcinoma patients undergoing treatment at The Third Affiliated Hospital of Harbin Medical University (Harbin, China), between January and June 2011, were enrolled into the study and underwent complete tumor evaluation. All patients provided written informed consent for the analysis. The study was approved by the ethics committee of the Third Affiliated Hospital of Harbin Medical University, Harbin, China. Clinical stages were determined according to the criteria of the American Joint Commission on Cancer (33) and the pathological type was diagnosed as lung adenocarcinoma. In addition, PB samples were obtained from 30 healthy patients who exhibited no evidence of any clinically detectable disease at the time of blood withdrawal.

### Processing of blood samples

To prevent the contamination of epithelial cells, a catheter was used and the first 5 ml of blood was discarded. Following this, a 5-ml sample of PB was obtained from patients prior to therapy. Sample processing was performed within 2 h of blood withdrawal. Mononuclear cells were enriched using density gradient centrifugation, which removed red blood cells and serum.

### Cell culture and processing blood-spiking samples

The lung adenocarcinoma cell line A549 was maintained in RPMI-1640 medium (Invitrogen, Carlsbad, CA, USA) and supplemented with 10% fetal bovine serum (Hyclone, Logan, UT, USA), penicillin (100 U/ml) and streptomycin (100 U/ml) at 37°C in a 5% CO_2_ air environment. For blood-spiking experiments, A549 cells were serially diluted in PBS and mixed with a 5-ml sample of PB which was obtained from one healthy patient to form concentrations of tumor cells of 1, 10, 100, 1,000 and 10,000 cells per 5-ml blood sample.

### Total RNA extraction and reverse transcription

Total RNA from mononuclear cells of the PB were extracted using TRIzol reagent (Invitrogen). RNA integrity was verified electrophoretically and quantified using UV spectrophotometry. The 260/280 ratio was 1.8–1.9. First strand cDNA was synthesized from total RNA using a first strand cDNA synthesis kit (Promega Corporation, Madison, WI, USA). The reverse transcription was performed in a reaction mixture consisting of AMV reverse transcriptase (high concentration), MgCl_2_ (25 mM), reverse transcription 10X buffer, dNTP mixture (10 mM), recombinant RNasin ribonuclease inhibitor and oligo(dT)_15_ primer. The reaction mixture, including 2 *μ*g RNA, was incubated at 70°C for 10 min, spun briefly and placed on ice. The 20-*μ*l reaction mixture was then incubated at 42°C for 45 min, heated to 95°C for 5 min and the resulting cDNA was stored at −20°C.

### Real-time PCR

Primers were designed for survivin, hTERT, CK-7 and TTF-1 using Premier 5.0 software (Palo Alto, CA, USA). The primers and Taqman probes were positioned to span exon-intron boundaries in order to reduce the detection of genomic DNA. The primer sequences and probes of each gene are shown in Table I. Real-time PCR and data collection were performed with a MiniOpticon™ system (Bio-Rad, CDF-3120, Hercules, CA, USA). The housekeeping gene, GAPDH, was used as an internal control to normalize the expression levels of survivin, hTERT, CK-7 and TTF-1. For the amplification of a total volume of 25 *μ*l, each reaction mixture contained 12.5 *μ*l PremixExTaq (DRR039S, Takara Bio, Inc., Shiga, Japan), 0.6 *μ*l forward primers (12.5 *μ*M), 0.6 *μ*l reverse primers (12.5 *μ*M), 0.3 *μ*l Taqman probe (5 *μ*M), 2 *μ*l cDNA and 9 *μ*l ddH_2_O. Amplification conditions are shown in Table II. For all samples, positive, negative and no template controls were performed. To ensure the reproducibility of results, all genes were tested in triplicate and the real-time PCR analyses were performed blinded for the identity and clinical outcome of the patients.

### Data analysis

Based on the three replications, mean crossing points (the beginning of the PCR exponential phase) were used in the calculations and the concentration of each tumor mRNA was normalized against that of GAPDH in each sample. If the mean Ct (threshold cycle) value for a gene of interest was ≥40, the gene expression was considered to be undetectable. Based on previous studies (11,34), the highest value of each marker in healthy patients was determined as a cut-off value, which was used to determine whether a patient’s PB sample was positive or not. For one marker, gene expression was regarded as positive when it was greater than the cut-off value. A PB sample was defined as CTC-positive (CTC^+^) if any of the four markers tested positive.

### Statistical analysis

All data were analyzed using the SPSS 17.0 software (Chicago, IL, USA). Chi-square analysis and Fisher’s exact test were used to analyze the correlation between the positive expression of the four markers and clinicopathological characteristics among lung adnocarcinoma patients and healthy patients. Bivariate correlations between variables were calculated by Spearman’s rank correlation coefficients. Patients’ survival curves were plotted by the Kaplan-Meier method and differences in survival were compared using the log-rank test. P<0.05 was considered to indicate a statistically significant result. Real-time PCR was calculated using linear regression and Pearson’s correlation.

## Results

### Sensitivity and specificity tests of real-time PCR

To confirm the sensitivity and specificity of our real-time RT-PCR assays, blood-spiking tests were performed to examine the number of detectable cells within PB samples. Serial numbers of human lung adenocarcinoma A549 cells were subjected to RNA extraction and real-time RT-PCR procedures. In our assay, the quantification of survivin, hTERT, CK-7 and TTF-1 was linearly correlated with the loading numbers of A549 cells in PB samples and the highest detection sensitivity observed was one A549 cell in 5 ml of PB.

**Figure 1:**
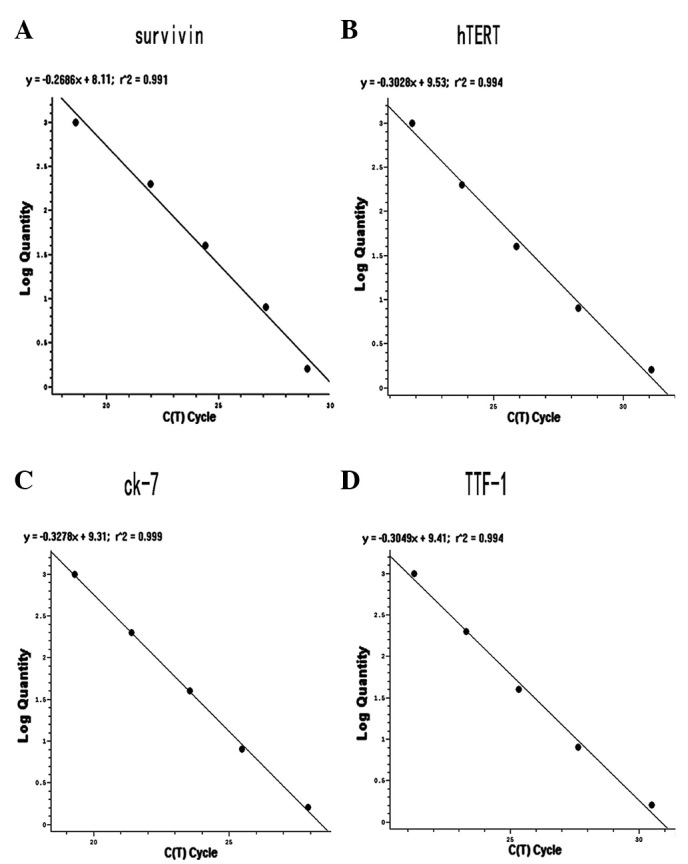
Standard curves for (A) survivin, (B) hTERT, (C) CK-7 and (D) TTF-1 estimation. Each curve was constructed using data from five external standards by plotting the Ct value against the input quantity of A549 cells (the concentration is 10,000 cells/5 ml, 1,000 cells/5 ml, 100 cells/5 ml, 10 cell/5 ml and 1 cell/5 ml. The concentration is represented from top to bottom in each figure) of the four markers. hTERT, human telomerase reverse transcriptase; TTF-1, thyroid transcription factor 1; CK-7, cytokeratin-7; Ct, threshold cycle.

Standard curves for the four markers were calculated with MiniOpticon system software (Fig. 1). The corresponding real-time PCR efficiency (E) of one cycle in the exponential phase was calculated according to the equation: E=10[−slope]−1 (35). Investigated transcripts demonstrated high real-time PCR efficiency rates which were detected using the Taqman probe. The efficiency of survivin, hTERT, CK-7 and TTF-1 was 85.6, 101, 108 and 102%, respectively. As shown in Fig. 1, the investigated concentration from 1 to 10000 cells/5 ml cDNA input appeared to have high linearity (Pearson correlation coefficient r>0.95).

### Relative expression of survivin, hTERT, CK-7 and TTF-1 mRNA in the PB of advanced lung adenocarcinoma patients

In our assay, the mRNA levels of these four markers were slightly amplified in PB samples of 30 healthy controls. To define a cut-off value for the normalization of mRNA expression of the four markers in the peripherial blood, the relative mRNA expression levels of each marker were examined in healthy patients. The highest values of survivin, hTERT, CK-7 and TTF-1 mRNA expression were 0.279, 0.0406, 0.094 and 1.182, respectively, which were equal to their expression levels in 0.4–11.8 A549 cells. Subsequently, four gene markers were compared with the highest value to determine the expression levels in PB samples of 68 lung adenocarcinoma patients. The sensitivity of survivin, hTERT, CK-7 and TTF-1 mRNA in patients was 41.18, 61.76, 41.18 and 35.29%, respectively. The sensitivity of the four markers combined was 82.35%. Compared with single marker detection, the sensitivity of the four markers in combination was significantly higher (Table III).

### Correlation of the mRNA expression levels of four markers with the clinicopathological features of advanced lung adenocarcinoma patients

Real-time PCR determination of the four marker mRNA expression levels was statistically analyzed to identify an association with the clinicopathological features of advanced lung adenocarcinoma patients. The characteristics of patients are summarized in Table IV. Survivin, hTERT and TTF-1 mRNA expression was significantly correlated with N classification (P=0.023, P=0.007 and P=0.007, respectively). Survivin, hTERT, CK-7 and TTF-1 mRNA expression was significantly correlated with distant metastases (P=0.031, P=0.001, P=0.031 and P=0.019, respectively). However, there was no significant association between the mRNA expression levels of the four markers and patients’ age or smoking (Table IV). Spearman correlation analysis demonstrated that high expression levels of survivin, hTERT and TTF-1 mRNA are positively correlated with lymph node classification, and survivin, hTERT, CK-7 and TTF-1 mRNA are positively correlated with distant metastasis (all r>0, P<0.05, data not shown).

### Disease progression and its association with CTC status

To evaluate whether the mRNA expression levels of the four markers in combination is associated with disease progression, we defined the PB sample from advanced lung adenocacinoma patients as CTC^+^ if any of the four markers tested positive. The rate of disease progression in the CTC^+^ group was 67.86% (38/56), while the rate of disease progression in the CTC^−^ group was 16.67% (2/12). There was a significant difference between the two groups (P=0.001), which was positively correlated with tumor progression (r=0.397, P<0.001). Meanwhile, we stratified the CTC^+^ group to a 1–2 marker mRNA overexpression group and a 3–4 marker mRNA overexpression group. Disease progression rate of the 3–4 marker mRNA overexpression group was 100% (18/18), while the 1–2 marker mRNA expression group was 52.63% (20/38). There was a significant difference between the two groups (P=0.000), which was positively correlated with tumor progression (r=0.474, P<0.000).

**Figure 2:**
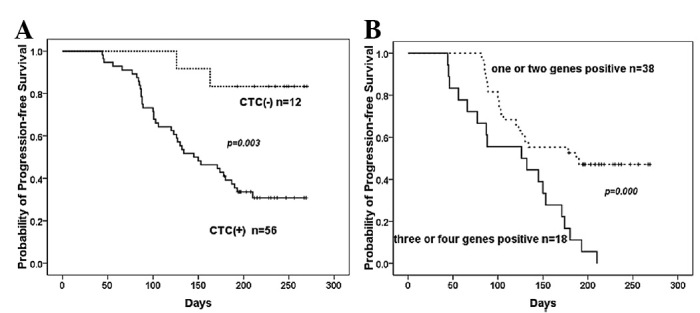
(A) Survivin, hTERT, CK-7 and TTF-1 overexpression correlated with disease progression (P=0.003). (B) With the increase in gene expression and increased risk of disease progression, progression-free survival is shorter (P=0.000). hTERT, human telomerase reverse transcriptase; TTF-1, thyroid transcription factor 1; CK-7, cytokeratin-7; Ct, threshold cycle.

The median progression time for the 68 cases was 150 days, with a range between 45 and 295 days. As shown in Fig. 2, compared with the CTC^−^ group, the CTC+ group had a shorter disease progression-free survival (P=0.003). In addition, in the 3–4 marker overexpression group, a shorter disease progression-free survival time was observed compared with the 1–2 marker expression group (P=0.000).

## Discussion

In this study, we developed a real-time RT-PCR assay to detect CTCs in advanced lung adenocarcinoma patients. To the best of our knowledge, this is the first time that survivin, hTERT, CK-7 and TTF-1 tumor-related mRNA markers have been used in combination to detect CTCs of advanced lung adenocarcinoma patients. This assay was designed to use Taqman probe to improve the specificity of the amplification products. In addition, the sensitivity demonstrated that target mRNAs are able to be detected at a sensitivity of one A549 cell in 5 ml of PB, which is sufficient to detect low levels of survivin, hTERT, CK-7 and TTF-1 mRNAs. Our present study demonstrates that the positive detection rates of survivin, hTERT, CK-7 and TTF-1 in the PB of advanced lung adenocarcinoma patients were 41.18, 61.76, 41.18 and 35.29%, respectively. The sensitivity of this multimarker combination was 82.35%, significantly higher than that of any single marker. Overexpression of survivin, hTERT, CK-7 and TTF-1 was significantly associated with distant metastasis and overexpression of survivin, hTERT and TTF-1 was significantly associated with N classification. In addition, the CTC^+^ group was inversely correlated with the disease progression of advanced lung adenocarcinoma patients.

Compared with the sampling of lymph nodes and bone marrow, PB collection is a noninvasive approach that may be conducted throughout the course of disease. A number of tumor-associated or epithelial-specific genes are used in the detection of CTCs of different types of cancer, including CK, Her2, CEA, MUC1, EpCAM, EGFR, hTERT, survivin, c-met, FN1 and several other mRNA markers (4,6,11,20). In our study, the tumor-associated genes survivin and hTERT, epithelial-specific gene CK-7 and lung or thyroid epithelial-specific TTF-1 were selected, and the positive detection rate of the four gene mRNAs ranged between 35.29 and 61.76%, which is similar to previous studies. These studies have demonstrated that the detection rate of circulating tumor-related mRNAs ranged between 30 and 60% in the PB of cancer patients (11,20). Since genes are expressed heterogeneously and the alternative expression of genes continuously occurs in tumor progression, no tumor marker was identified to be consistently and specifically expressed in all tumor cases. Single marker detection may limit the reliability of the assay. Consequently, we combined the four markers to detect levels of CTCs in advanced lung adenocarcinoma patients. The sensitivity of the four markers in combination was 82.35%, an increase of 10.35% compared with results from the study by Sher *et al,* detecting a combination of four marker genes (36). We demonstrated that, compared with single marker detection, combined use of the four mRNA markers is capable of improving the sensitivity of CTC detection in the PB of patients with advanced lung adenocarcinoma. These results suggest that the use of multiple markers is able to compensate for tumor cell heterogeneity in marker expression, low mRNA levels and the rarity of CTCs in the PB.

Furthermore, we have analyzed the correlation between these four mRNA markers with the clinicopathological features of advanced lung adenocarcinoma. We identified that the over-expression of survivin, hTERT and TTF-1 mRNA is positively correlated with lymph node classification, and overexpression of survivin, hTERT, CK-7 and TTF-1 mRNA is positively correlated with distant metastasis. The present study strongly suggests that the four gene mRNA marker combination may provide a valuable tool to identify subsets of advanced lung adenocarcinoma patients with more aggressive tumors, which have a high risk of metastasis and recurrence. These results are consistent with the majority of other studies of lung and breast cancer (7,11,20). Following 10 months of follow-up, disease progression-free survival was also significantly shorter in patients with CTC^+^ compared with those with CTC^−^, and with the increased number of genes expressed, and increased risk of disease progression, the progression-free survival shortened. These results suggest that survivin, hTERT, CK-7 and TTF-1 mRNA are important in lung adenocarcinoma development and analysis of the four marker genes may provide valuable prediction information of disease progression in patients. In terms of overall survival, the follow-up period of the present study was only 10 months, which is too short a time to assess the values of the investigated CTC markers as predictors of overall survival. A longer observation time with more serial monitoring is necessary to validate the potential usefulness of these four markers in combination as an overall predictor of survival.

In conclusion, we used quantitative real-time RT-PCR to detect survivin, hTERT, CK-7 and TTF-1 mRNA expression levels in PB samples of advanced lung adenocarcinoma patients. We identified four mRNA markers that were capable of significantly improving the sensitivity of detecting CTCs compared with single marker assays. Multiple marker expression is positively correlated with N classification and distant metastasis. Multiple marker-positive CTCs are a useful surrogate predictor of disease progression. However, it needs to be studied in larger patient cohorts including early stage patients to precisely define the clinical relevance of the four mRNA markers.

## Figures and Tables

**Table I:** List of all primers used for PCR.

Primer	5′-3′ sequence	Size of product (bp)
Survivin (sense)	AAGAACTGGCCCTTCTTGGA	253
Survivin (antisense)	CAACCGGACGAATGCTTTT	
Survivin Taqman probe	CCAGATGACGACCCCATTGGGCCGG	
hTERT (sense)	TACGTCGTGGGAGCCAGAAC	86
hTERT (antisense)	TTCCGCAGAGAAAAGAGGGCCGA	
hTERT Taqman probe	TTCCGCAGAGAAAAGAGGGCCGA	
CK-7 (sense)	GACATCGAGATCGCCACCTAC	162
CK-7 (antisense)	ATTGCTGCCCATGGTTCCC	
CK-7 Taqman probe	AATGCCACCGCCACTGCTACTGCC	
TTF-1 (sense)	CTTCGCCTTCCCCCTCTCC	156
TTF-1 (antisense)	CCCTCCATGCCCACTTTCTTG	
TTF-1 Taqman probe	TCTTCCTTCCTCCTCCAGCCGCCG	
GAPDH (sense)	GAAGGTGAAGGTCGGAGTC	225
GAPDH (antisense)	GAAGATGGTGATGGGATTTC	
GAPDH Taqman probe	CAAGCTTCCCGTTCTCAGCC	

hTERT, human telomerase reverse transcriptase; TTF-1, thyroid transcription factor 1; CK-7, cytokeratin-7.

**Table II:** Real-time PCR amplification conditions.

Marker	Pre-denaturation	Denaturation	Annealing	Extension	Cycles
Survivin	95°C (2 min)	95°C (5 sec)	55°C (20 sec)	72°C (20 sec)	40
hTERT	95°C (5 min)	95°C (20 sec)	56°C (30 sec)	72°C (30 sec)	40
CK-7	95°C (2 min)	95°C (15 sec)	58°C (30 sec)	72°C (30 sec)	40
TTF-1	95°C (2 min)	95°C (15 sec)	58°C (30 sec)	72°C (30 sec)	40

Values represent temperature and length of time. hTERT, human telomerase reverse transcriptase; TTF-1, thyroid transcription factor 1; CK-7, cytokeratin-7.

**Table III:** Positive detection rates for four gene markers in the peripheral blood of patients with advanced lung adenocarcinoma (n=68).

	Survivin	hTERT	CK-7	TTF-1	Combined
Above cut-off (n)	28	42	28	24	56
Below cut-off (n)	40	26	40	44	12
Positive rate (%)	41.18	61.76	41.18	35.29	82.35

Combined signifies positive if any of the four markers tested positive. hTERT, human telomerase reverse transcriptase; TTF-1, thyroid transcription factor 1; CK-7, cytokeratin-7.

**Table IV:** Correlation between clinicopathological features of advanced lung adenocarcinoma patients and the expression of mRNA markers.

	Survivin (%)			hTERT (%)			CK-7 (%)			TTF-1 (%)		
			
Characteristic	Positive	Negative	P-value	Positive	Negative	P-value	Positive	Negative	P-value	Positive	Negative	P-value
Age (years)												
≤50	6 (60.0)	4 (40.0)		4 (40.0)	6 (60.0)		6 (60.0)	4 (60.0)		6 (60.0)	4 (60.0)	
>50	22 (37.9)	36 (62.1)	0.190^a^	38 (65.5)	20 (34.5)	0.125^a^	22 (37.9)	36 (62.1)	0.190^a^	18 (31.0)	40 (69.0)	0.077^a^
Smoking												
No	16 (44.4)	20 (55.6)		20 (55.6)	16 (44.4)		12 (33.3)	24 (66.7)		14 (38.9)	22 (61.1)	
Yes	12 (37.5)	20 (62.5)	0.635	22 (68.8)	10 (31.3)	0.264	16 (50.0)	16 (50.0)	0.163	10 (31.3)	22 (68.8)	0.511
N classification												
N0	7 (25.0)	21 (75.0)		12 (42.9)	16 (57.1)		8 (28.6)	20 (71.4)		4 (14.3)	24 (85.7)	
N1–3	21 (52.5)	19 (47.5)	0.023	30 (75.0)	10 (25.0)	0.007	20 (50.0)	20 (50.0)	0.077	20 (50.0)	20 (50.0)	0.002
Distant metastases												
No	8 (26.7)	22 (73.3)		12 (40.0)	18 (60.0)		8 (26.7)	22 (73.3)		6 (20.0)	24 (80.0)	
Yes	20 (52.6)	18 (47.4)	0.031	30 (78.9)	8 (21.1)	0.001	20 (52.6)	18 (47.4)	0.031	18 (47.4)	20 (52.6)	0.019

Cut-off values of the four markers survivin, hTERT, CK-7 and TTF-1 are 0.279, 0.0406, 0.094 and 1.182, respectively.

a P-values were obtained by Chi-square analysis or Fisher’s exact test. hTERT, human telomerase reverse transcriptase; TTF-1, thyroid transcription factor 1; CK-7, cytokeratin-7.

## Abbreviations:

CTCs: circulating tumor cells
PB: peripheral blood

## References

[1] Parkin DM, Pisani P, Ferlay J (1999). Global cancer statistics. CA Cancer J Clin.

[2] Hoffman PC, Mauer AM, Vokes EE (2000). Lung cancer. Lancet.

[3] Allard WJ, Matera J, Miller MC (2004). Tumor cells circulate in the peripheral blood of all major carcinomas but not in healthy subjects or patients with nonmalignant diseases. Clin Cancer Res.

[4] Tewes M, Aktas B, Welt A (2009). Molecular profiling and predictive value of circulating tumor cells in patients with metastatic breast cancer: an option for monitoring response to breast cancer related therapies. Breast Cancer Res Treat.

[5] Schuster R, Bechrakis NE, Stroux A (2007). Circulating tumor cells as prognostic factor for distant metastases and survival in patients with primary uveal melanoma. Clin Cancer Res.

[6] Iinuma H, Okinaga K, Egami H (2006). Usefulness and clinical significance of quantitative real-time RT-PCR to detect isolated tumor cells in the peripheral blood and tumor drainage blood of patients with colorectal cancer. Int J Oncol.

[7] Devriese LA, Bosma AJ, van de Heuvel MM, Heemsbergen W, Voest EE, Schellens JH (2012). Circulating tumor cell detection in advanced non-small cell lung cancer patients by multi-marker QPCR analysis. Lung Cancer.

[8] Moreno JG, Miller MC, Gross S, Allard WJ, Gomella LG, Terstappen LW (2005). Circulating tumor cells predict survival in patients with metastatic prostate cancer. Urology.

[9] Cristofanilli M, Mendelsohn J (2006). Circulating tumor cells in breast cancer: Advanced tools for ‘tailored’ therapy?. Proc Natl Acad Sci USA.

[10] Nagrath S, Sequist LV, Maheswaran S (2007). Isolation of rare circulating tumour cells in cancer patients by microchip technology. Nature.

[11] Yoon SO, Kim YT, Jung KC, Jeon YK, Kim BH, Kim CW (2011). TTF-1 mRNA-positive circulating tumor cells in the peripheral blood predict poor prognosis in surgically resected non-small cell lung cancer patients. Lung Cancer.

[12] Zieglschmid V, Hollmann C, Böcher O (2005). Detection of disseminated tumor cells in peripheral blood. Crit Rev Clin Lab Sci.

[13] Sieuwerts AM, Kraan J, Bolt-de Vries J (2009). Molecular characterization of circulating tumor cells in large quantities of contaminating leukocytes by a multiplex real-time PCR. Breast Cancer Res Treat.

[14] Ambrosini G, Adida C, Altieri DC (1997). A novel anti-apoptosis gene, survivin, expressed in cancer and lymphoma. Nat Med.

[15] Blanc-Brude OP, Mesri M, Wall NR, Plescia J, Dohi T, Altieri DC (2003). Therapeutic targeting of the survivin pathway in cancer: initiation of mitochondrial apoptosis and suppression of tumor-associated angiogenesis. Clin Cancer Res.

[16] Pennati M, Folini M, Zaffaroni N (2007). Targeting survivin in cancer therapy: fulfilled promises and open questions. Carcinogenesis.

[17] Adida C, Berrebi D, Peuchmaur M, Reyes-Mugica M, Altieri DC (1998). Anti-apoptosis gene, survivin, and prognosis of neuroblastoma. Lancet.

[18] Bertazza L, Mocellin S, Marchet A (2009). Survivin gene levels in the peripheral blood of patients with gastric cancer independently predict survival. J Transl Med.

[19] Cao W, Yang W, Li H (2011). Using detection of survivin-expressing circulating tumor cells in peripheral blood to predict tumor recurrence following curative resection of gastric cancer. J Surg Oncol.

[20] Shen C, Hu L, Xia L, Li Y (2009). The detection of circulating tumor cells of breast cancer patients by using multimarker (Survivin, hTERT and hMAM) quantitative real-time PCR. Clin Biochem.

[21] Daniel M, Peek GW, Tollefsbol TO (2012). Regulation of the human catalytic subunit of telomerase (hTERT). Gene.

[22] Miura N, Horikawa I, Nishimoto A (1997). Progressive telomere shortening and telomerase reactivation during hepatocellular carcinogenesis. Cancer Genet Cytogenet.

[23] Wu CH, Lin SR, Hsieh JS (2006). Molecular detection of disseminated tumor cells in the peripheral blood of patients with gastric cancer: evaluation of their prognostic significance. Dis Markers.

[24] Chang YL, Lee YC, Liao WY, Wu CT (2004). The utility and limitation of thyroid transcription factor-1 protein in primary and metastatic pulmonary neoplasms. Lung Cancer.

[25] Lazzaro D, Price M, de Felice M, Di Lauro R (1991). The transcription factor TTF-1 is expressed at the onset of thyroid and lung morphogenesis and in restricted regions of the foetal brain. Development.

[26] Pelosi G, Fraggetta F, Pasini F (2001). Immunoreactivity for thyroid transcription factor-1 in stage I non-small cell carcinomas of the lung. Am J Surg Pathol.

[27] Wicha MS (2006). Cancer stem cells and metastasis: lethal seeds. Clin Cancer Res.

[28] Kalekou H, Miliaras D (2011). Cytokeratin 7 and 20 expression in gallbladder carcinoma. Pol J Pathol.

[29] Kawaguchi K, Shin SJ (2012). Immunohistochemical staining characteristics of low-grade adenosquamous carcinoma of the breast. Am J Surg Pathol.

[30] Coban S, Ormeci N, Savaş B (2012). Evaluation of Barrett’s esophagus with CK7, CK20, p53, Ki67, and cyclooxygenase expressions using chromoendoscopical examination. Dis Esophagus.

[31] Ross H, Martignoni G, Argani P (2012). Renal cell carcinoma with clear cell and papillary features. Arch Pathol Lab Med.

[32] Xi L, Nicastri DG, El-Hefnawy T, Hughes SJ, Luketich JD, Godfrey TE (2007). Optimal markers for real-time quantitative reverse transcription PCR detection of circulating tumor cells from melanoma, breast, colon, esophageal, head and neck, and lung cancers. Clin Chem.

[33] Greene FL, Page DL, Fleming ID, Fritz AG, Balch CM, Haller DG, Morrow M (2001). AJCC Cancer Staging Handbook.

[34] Tjensvoll K, Oltedal S, Farmen RK (2010). Disseminated tumor cells in bone marrow assessed by TWIST1, cytokeratin 19, and mammaglobin A mRNA predict clinical outcome in operable breast cancer patients. Clin Breast Cancer.

[35] Bustin SA, Benes V, Garson JA (2009). The MIQE guidelines: minimum information for publication of quantitative real-time PCR experiments. Clin Chem.

[36] Sher YP, Shih JY, Yang PC (2005). Prognosis of non-small cell lung cancer patients by detecting circulating cancer cells in the peripheral blood with multiple marker genes. Clin Cancer Res.

